# Investigation of the Interfacial Fusion Bonding on Hybrid Additively Manufactured Components under Torsional Load

**DOI:** 10.3390/polym16192719

**Published:** 2024-09-26

**Authors:** Melike Kizak, Anna von Bartschikowski, Anna Trauth, Christian Heigl, Klaus Drechsler

**Affiliations:** 1 Chair of Carbon Composites, Technical University of Munich, Boltzmannstr. 15, 85748 Garching, Germany; anna.bartschikowski@tum.de (A.v.B.); klaus.drechsler@tum.de (K.D.); 2 Institute of Materials Resource Management, University of Augsburg, Am Technologiezentrum 8, 86159 Augsburg, Germany; anna.trauth@mrm.uni-augsburg.de; 3 Toray Automotive Center Europe, Am Gfild 6, 85375 Neufahrn bei Freising, Germany; christian.heigl.t8@mail.toray

**Keywords:** composites, additive manufacturing, fused filament fabrication, hybrid materials, injection molding, interface bonding, mechanical characterization, torsion

## Abstract

Hybrid manufacturing processes integrate multiple manufacturing techniques to leverage their respective advantages and mitigate their limitations. This study combines additive manufacturing and injection molding, aiming to efficiently produce components with extensive design flexibility and functional integration. The research explores the interfacial fusion bonding of hybrid additively manufactured components under torsional loading. Specifically, it examines the impact of various surface treatments on injection molded parts and the influence of different build chamber temperatures during additive manufacturing on torsional strength. Polycarbonate components, neat, with glass or carbon fiber-reinforcement, are produced and assessed for dimensional accuracy, torsional strength, and fracture behavior. The findings emphasize the critical role of surface treatment for the injection molded components before additive manufacturing. Additionally, the study identifies the influence of chamber temperatures on both dimensional accuracy and torsional strength. Among all investigated materials, plasma-treated neat samples exhibited the best torsional strength. The torsional strength was increased by up to 87% by actively heating the build chamber to 186 °C for neat polycarbonate. These insights aim to advance the quality and performance of hybrid additively manufactured components, broadening their application potential across diverse fields.

## 1. Introduction

Hybrid manufacturing utilizes multiple manufacturing techniques to produce a component, strategically leveraging the advantages of each method. Combining additive manufacturing (AM) and injection molding (IM) opens new possibilities for producing complex parts and has gained increasing importance. This combination allows for the productivity and precision of IM to be paired with the design freedom of AM. In recent years, hybrid manufacturing processes that combine AM and IM have garnered interest across various industries [1,2].

In integrating AM structures as inserts in IM processes, complex or lightweight structures are created using AM and placed into IM tools before injecting liquid material. These inserts can enhance the strength and stiffness of the final IM component without significantly increasing its overall weight [3]. Additionally, hybrid manufacturing methods can be employed to repair existing parts, where defective or worn-out sections are repaired or supplemented with metal or plastic, enabling the reuse of components with economic and environmental benefits [4,5,6,7].

Another application area is printing on IM parts with structurally optimized or functionally integrated features. AM techniques are used to add additional structures to already molded parts, referred to as substrates. These AM structures can include reinforcements [8], cooling channels, or sensors integrated into the component [9,10], enabling the production of complex parts that are both lighter and more functional [11].

Regardless of the application and production process, the bonding between the IM part and the AM structure is crucial for the quality of the manufacturing process [12,13]. To understand how the printed material adheres to the substrate, the interactions at the material surfaces must be considered, primarily involving adhesion and diffusion processes. A high adhesion strength between the substrate surface and the first printed layer correlates with good wettability [14]. A well-wettable surface achieves a larger contact area between the plastic melt and the solid surface, promoting diffusion processes at the interface. During the printing process, the surfaces of the substrate and the molten filament each possess a specific energy due to the free bonds on the surfaces. The stronger these free bonds, the higher the surface energy of the materials and the greater the tendency for adhesion, resulting in better bonding. Thus, higher surface energy leads to better adhesion between the AM and the IM part. Good adhesion can only be achieved if molecular segments at the respective surfaces of the substrate and AM structure interact, requiring a similarity in the strength and nature of the molecular forces at both surfaces [15,16].

The energy of plastics is structurally low [15], but wettability can be improved by increasing surface energy, as seen in plasma treatments. Plasma, an ionized gas consisting mainly of positively charged ions and free electrons, can split chemical bonds on the surface and create new functional groups, altering the surface’s chemical properties [17,18].

Penter et al. [12] demonstrated the effectiveness of plasma using modified tensile tests, where a plasma-treated IM plate was subsequently printed using the fused filament fabrication process. A smaller contact angle indicated increased surface energy, confirmed by higher mechanical properties in the tensile test.

The second phenomenon relevant to bonding between the IM part and the AM structure is diffusion [19]. Plastics already possess a certain permeability for liquids and gases due to the free volume between macromolecules. When plastics are heated, the mobility of the molecules increases, allowing adjacent molecules of the plastic melt to diffuse across the interface. The exact diffusion rate depends on various factors, such as temperature and time. Better interfacial bonding between the IM part and the AM structure can be achieved by increasing the build chamber temperature, thus enhancing molecular mobility and slowing the cooling of the extruded melt, giving the molecules more time to diffuse [20,21,22].

Various mechanical tests can be employed to characterize the adhesion between two layers. According to Grellmann and Seidler [23], these tests can be categorized based on the type of stress applied: tensile, compressive, bending, torsional, and shear stress. This work focuses exclusively on torsional stress.

In the study by Gong et al. [24], a hybrid manufacturing process combining AM and IM was investigated to improve the mechanical properties of acrylonitrile butadiene styrene (ABS) samples. Additively manufactured ABS inserts were placed in a T-shaped injection mold before the casting process. The study found that hybrid samples, especially those with a T-shape and a medium filling density of 50%, exhibited improved strength and potential cost savings in producing customized products.

Moritzer et al. [8] aimed to enhance the strength and stiffness of thin-walled plastic components. These components often have poor mechanical properties, so the thin-walled areas were reinforced with specially adapted structures using fused filament fabrication. The material used was the high-performance thermoplastic polyetherimide. Tensile, compressive, bending, and torsion tests were conducted to determine the static strength properties. The resulting hybrid structure exhibited higher strength or stiffness depending on the reinforcement structure’s shape compared to components without reinforcement structures. While following existing DIN standards for tensile, compressive, and bending tests, they developed a unique approach for torsion testing, fixing the test specimen in a special device and loading it in a screw testing machine until failure.

Weaver et al. [25] characterized the interface of a hybrid component by the first part being wrought and then building the second part using AM. Although a powder-bed-based method was used instead of filament printing, interlayer adhesion remained crucial. The test specimens had threaded ends to apply torque during torsion testing, revealing that the additive part generally had higher strength and lower ductility than the rolled specimens. No failure occurred at or near the interface between the rolled substrate and the AM material, highlighting the potential of AM for adding features or repairing existing structures.

The study by Guo et al. [26] explores the use of ultrasonic additive manufacturing to create high-strength joints between carbon-fiber-reinforced polymer and aluminum alloy for vehicle structures. The process enabled the embedding of carbon fibers into an aluminum matrix, resulting in improved mechanical performance. The hybrid structures were tested in four-point bending, dynamic axial crush, and quasi-static torsion tests. The results demonstrated that UAM-based joints exhibit 13% higher peak torque and fail by a gradual shearing of the interface and thus allow structural integrity compared to conventional riveted joints.

## 2. Research Question

Despite the extensive research on hybrid AM, there remains a significant gap in the fundamental understanding of the adhesion mechanisms between AM structures and IM parts. The previous studies assume good adhesion between the components but have not isolated the adhesion scenario. The systematic investigation of interface bonding between AM and IM parts is crucial. This research aims to address this gap by conducting a detailed and systematic investigation of the interface bonding between AM and IM parts. Unlike previous studies, which have largely focused on the overall performance of hybrid components, this work isolates the adhesion process to uncover the underlying mechanisms that govern the strength and reliability of the bond. Therefore, this research provides fundamental insights into the adhesion mechanisms, which are crucial for enhancing the reliability and performance of hybrid manufactured components. These insights not only contribute to the existing body of knowledge but also enable the development of innovative solutions for a wide range of applications, thereby advancing the field of hybrid manufacturing.

This study investigates the influence of various surface treatments on the IM substrate and the effect of different build chamber temperatures during AM. The primary focus is on how these factors impact the strength and adhesion quality between the IM and AM layers by means of torsion tests and fracture analysis. The specific research questions addressed in this study are:

- How do different surface treatments of the IM substrate affect the torsional strength of the hybrid component?
- How do varying chamber temperatures during hybrid manufacturing affect the torsional strength of the hybrid component?
- How do these factors influence the fracture patterns observed in torsion tests?

The study involves a series of torsion tests to evaluate the adhesion quality under different conditions, followed by a detailed analysis of the resulting fracture patterns to understand the failure mechanisms at the interface.

## 3. Materials and Methods

### 3.1. Materials

This study used three material combinations: polycarbonate (PC) printed segment onto PC IM substrate, glass-fiber-reinforced PC (G-PC) printed segment onto G-PC IM substrate, and carbon-fiber-reinforced PC (C-PC) printed segment onto C-PC IM substrate. The PC IM substrate plates were produced from XANTAR 18 UR-PC granulate provided by Mitsubishi Engineering-Plastics Corporation (Minato City, Japan), while the printed segments were created using PolyLite PC filament from Polymaker (Changshu, China), with a diameter of 1.75 mm. DAHLTRAM C-250GF granulate from Airtech Europe Sarl (Differdingen, Luxemburg), which contains 20% glass fiber, was utilized for the G-PC substrate plates. Ultrafuse PC GF30 filament from BASF (Ludwigshafen, Germany), featuring a 1.75 mm diameter, was used for the printing. The C-PC substrate plates were made using DAHLTRAM C-250CF granulate from Airtech, which has a 20% carbon fiber content, and the AM structures were produced with CarbonX Fiber ezPC filament, also 1.75 mm in diameter, from 3DXTech (Grand Rapids, MI, USA).

### 3.2. Differential Scanning Calorimetry (DSC)

Prior to sample fabrication, the base materials were analyzed using DSC. A DSC Q200 device and an RCS90 cooling unit from TA Instruments (New Castle, DE, USA), were employed to determine the materials’ glass transition temperature ($T_{g}$). This temperature is crucial for sample production as the experimental series investigated manufacturing at various build chamber temperatures in relation to $T_{g}$. Two filament samples and two granulate samples were analyzed for each material type (PC, G-PC, and C-PC). Each sample underwent two heating cycles from 30 °C to 200 °C at a rate of 10 K/min. Only the second cycle was used for evaluation, as it eliminated effects such as residual stresses or moisture in the material. $T_{g}$ was determined according to DIN EN ISO 11357-2 [27].

### 3.3. Sample Preparation

Before manufacturing the samples, it was necessary to dry both the granulate for the IM substrate parts and the filaments for the printed segment. A Memmert UF 110 Plus universal oven was used for this purpose, and it was also employed to dry the three granulates before IM and the plates directly before printing. As recommended by the manufacturers, the drying process was carried out at 120 °C for at least four hours.

The substrate plates were manufactured using an ENGEL (Schwertberg, Austria), tie-bar-less injection molding machine VC 330/90 tech. The injection unit was equipped with an all-purpose 35 mm screw, heated by four independent zones, with a temperature range of 315 °C to 330 °C from the feeder to the nozzle. The nozzle itself was maintained at a constant temperature of 315 °C. Following the injection of molten material into the heated tool at a temperature of 105 °C, the injection unit applied back pressure for a period of 25 s until the injection gate was frozen. The back pressure was 700 bar. Subsequently, the injection unit plasticized a second shot, which was then cooled for 8 s. During this time, the injection unit detached from the tool, enabling the tool to open and the press side to eject the plate. Thereafter, the sprue was removed using a band saw to prepare the plates for the subsequent AM process.

To investigate the influence of different surface treatments on the substrate plates’ interfacial bonding, the substrate plates underwent sandblasting, manual sanding, and plasma treatment, with untreated plates serving as a reference.

For sandblasting, a Sandmaster AG (Zofingen, Switzerland) machine was used with glass beads sized between 90μm and 150μm as the abrasive medium, applied at a pressure of 3 bar. Manual sanding was performed using 180-grit sandpaper, involving circular motions with even pressure until a visually homogeneous roughness was achieved. These processes aimed to increase surface roughness by removing material from the plates’ surfaces. After the respective surface treatment, each sample was cleaned with compressed air and isopropanol to remove any abrasive residues and ensure precise measurement of the achieved surface roughness. The final method, plasma treatment, employed the piezobrush PZ3 plasma pen from relyon plasma GmbH. The standard module, suitable for non-conductive substrates like plastics, treated the PC and G-PC plates, while the nearfield module, designed for conductive materials, was used for the C-PC plates. The plasma treatment was manually conducted by holding the plasma pen at full intensity over the area where an AM sample would later be printed for 30 s per interface. In this case, the substrate plates were cleaned with isopropanol before the plasma treatment.

Moreover, four different chamber temperatures were examined: no active tempering ($T_{0}$), $T_{g}$ ($T_{1}$), $T_{g}$ + 20 °C ($T_{2}$), $T_{g}$ + 40 °C ($T_{3}$).

**Figure 1:**
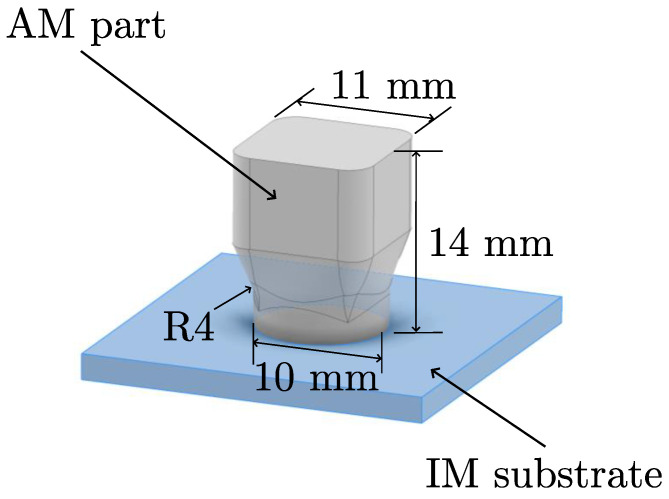
Geometry of the hybrid sample.

The test specimens were fabricated using the filament printer GEWO Performer 260 (Woerth/Hoerlkofen, Germany). The slicing software Simplify3D (version 4.1.2) was used to prepare the print files. The specific printing parameters are listed in Table 1. The geometry of the specimens, with a height of 11 mm and base diameter of 10 mm, is shown in Figure 1.

### 3.4. Roughness Measurement and Fracture Behavior

To determine the average surface roughness ($R_{a}$) of both treated and untreated injection-molded plates, as well as for the subsequent analysis of fracture patterns and their maximal roughness value $R_{z}$, a Keyence VR-5000 profilometer (Osaka, Japan) was used. Multiple line roughness measurements were taken in both vertical and horizontal directions along 22 lines on three randomly selected sample plates.

### 3.5. Dimensional Accuracy

To assess the accuracy of the printing process, all printed specimens were measured once using a digital caliper with an accuracy of ±0.03 mm. The measurements included the height of the specimens and the diameter of the first printed layers.

### 3.6. Torsion Test

**Figure 2:**
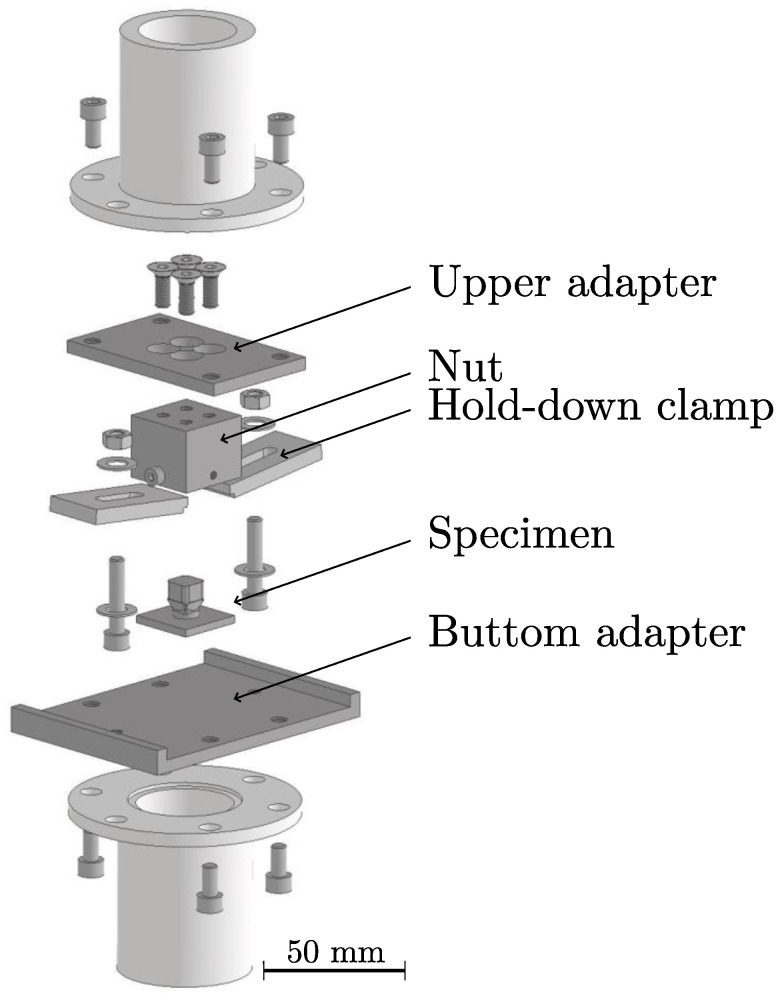
Torsion test set-up.

The torsion tests were conducted using the ElectroPuls E10000 Linear-Torsion machine from Instron GmbH (Darmstadt, Germany), equipped with a load cell capable of measuring up to 25 Nm. The tests were performed using the manufacturer’s WaveMatrix 2 materials testing software. A pre-load tensile force of 1 N was applied. The AM part of the hybrid specimen was rotated at a rate of 1°/s until it reached an angle of $360^{\circ }$ or until the test was manually stopped upon failure. Five samples per configuration were tested. Figure 2 shows the test setup, including the adapter.

## 4. Results

### 4.1. DSC

Table 2 presents the arithmetic mean of $T_{g}$ for each material (PC, G-PC, and C-PC) and material form (substrate and filament). A comparison of the average $T_{g}$ between granulate and filament of the same material reveals a maximum difference of 35 °C for PC, while the difference for G-PC is 7 °C and 3 °C for C-PC.

### 4.2. Roughness Measurement

**Figure 3:**
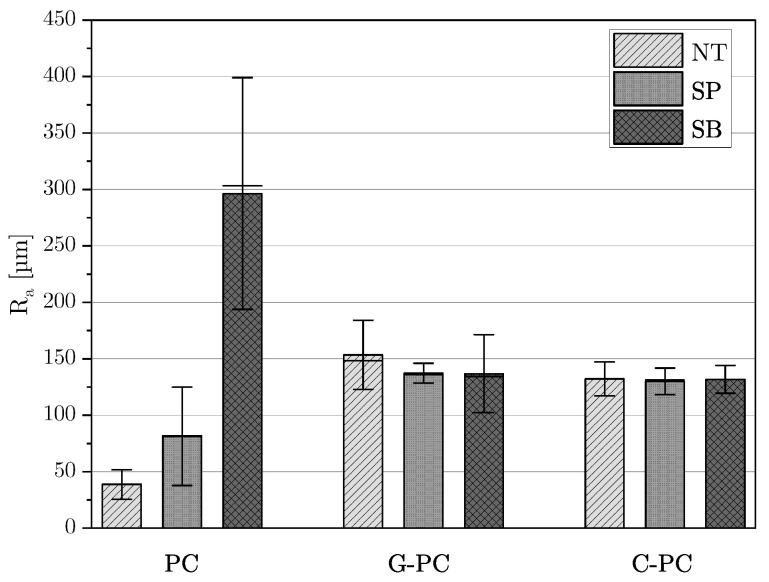
Average roughness value $R_{a}$ of PC, G-PC, and C-PC for surface treatments no treatment (NT), sandpaper (SP), and sandblasting (SB).

The results of the surface roughness analysis, categorized by material and surface treatment, are displayed in Figure 3. The arithmetic mean of the $R_{a}$ values and the standard deviation are shown. Untreated PC plates had the lowest roughness at 38.6μm. Manual sanding increased the roughness of PC substrates to 81.4μm, and sandblasting further increased it to 296.4μm. An opposite trend was observed for fiber-reinforced plates: untreated plates had the highest roughness. Surface treatments reduced roughness, with G-PC substrates showing a reduction of 10.6% after manual sanding and 10.9% after sandblasting. For C-PC plates, sanding resulted in a decrease of 1.7%, and sandblasting led to a decrease of 0.3%.

### 4.3. Dimensional Accuracy

**Figure 4:**
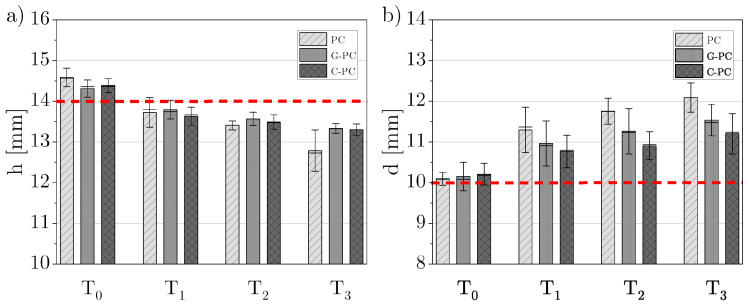
(**a**) Height *h* and (**b**) base diameter *d* measurements of PC, G-PC, and C-PC for chamber temperatures $T_{0}$–$T_{3}$ with the target dimensions of 14 mm for the height and 10 mm for the base diameter marked as dashed red lines.

The measurement results of the height and base diameter of the printed segment are graphically presented in Figure 4. The red dotted horizontal line indicates the target dimension of 14 mm for the height and 10 mm for the base diameter. For all materials, it was observed that height decreased and base diameter increased with rising build chamber temperature. Samples of the same material printed at the same build chamber temperature showed consistently low variations, regardless of the surface treatment.

For PC, the highest geometric accuracy in height was observed at $T_{g}$. Deviations from the target value were −2.6% for PC, −1.0% for G-PC, and −2.7% for C-PC. An unheated build chamber provided the second-best accuracy for PC and C-PC, while for G-PC, a build chamber temperature 20 °C above $T_{g}$ was optimal. The lowest heights for all materials were recorded at 40 °C above $T_{g}$, with deviations of −8.7% for PC, −4.8% for G-PC, and −4.9% for C-PC. Overall, the fiber-reinforced samples showed smaller deviations compared to the neat PC samples.

Regarding the diameter of the first printed layers, the highest accuracy was achieved in an unheated build chamber, with accuracy decreasing as the temperature increased. The smallest deviation for PC was +0.9%, for G-PC +1.3%, and for C-PC +2.1%. PC samples consistently exhibited the highest deviation at higher temperatures, while C-PC samples had the smallest deviation among the materials tested.

### 4.4. Torsion Test

The results of the torsion tests are presented in Table 3, Table 4 and Table 5. The lowest torsional stress and failure angle in PC samples were observed for samples printed in an unheated chamber. The failure angle of the samples for all treatments increased until chamber temperature $T_{2}$. Untreated and plasma-treated samples printed at $T_{2}$ showed the highest torsional stress before decreasing for samples printed at $T_{3}$. The samples with sandblasted and sanded PC plates showed an increase in strength for samples printed up to $T_{1}$, followed by a decrease for samples printed up to $T_{3}$.

Untreated, plasma-, and sandpaper-treated G-PC samples showed maximal torsional stress for samples printed at $T_{3}$. The samples with the sandblasted G-PC substrate exhibit the maximal strength printed with chamber temperature $T_{2}$. Higher chamber temperatures reduced the results’ scatter. The fracture angles for G-PC samples also increased with chamber temperature, from an average of $4.30^{\circ }$ for samples printed in an unheated chamber to $31.35^{\circ }$ for samples printed at the highest temperature.

The C-PC samples’ lowest strength and failure angle were also observed at $T_{0}$. The maximal failure angle for all treatments was at chamber temperature $T_{3}$. The samples with untreated and plasma-treated substrates showed the highest torsional stress at $T_{3}$, while sandpaper-treated and sandblasted samples peaked at $T_{2}$.

### 4.5. Fracture Behavior

**Figure 5:**
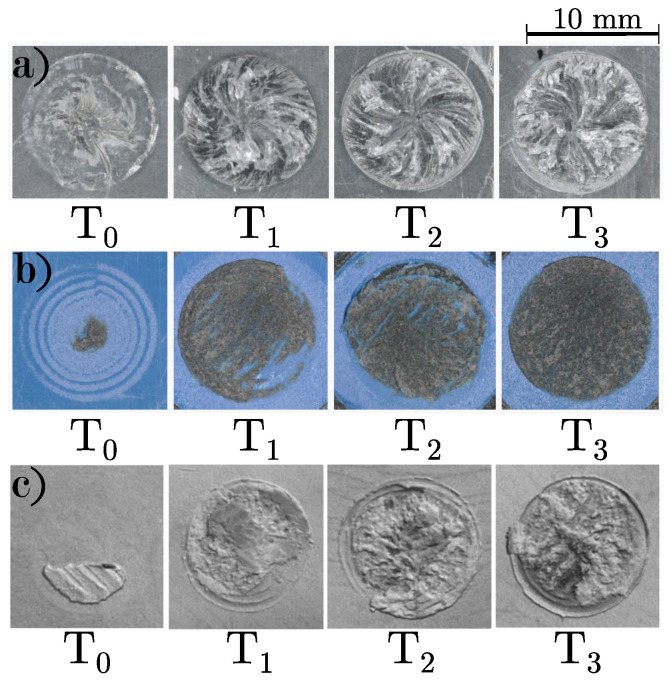
Representative fracture surface of plasma-treated (**a**) PC, (**b**) G-PC, and (**c**) C-PC for chamber temperatures $T_{0}$–$T_{3}$.

Figure 5 shows representative fracture surfaces of plasma-treated substrates arranged from left to right in order of increasing build chamber temperature. The fracture surface roughness for all materials increased with rising build chamber temperature. In PC samples, a spiral shape was visible on the fracture surfaces. The maximal roughness value $R_{z}$ of the respective circular fracture surface are shown in Table 6. It is evident that the roughness consistently increases with temperature, being highest in PC and lowest in C-PC. While the roughness from $T_{0}$ to $T_{1}$ increases approximately two to four times, the difference among the three higher temperatures is comparatively small.

## 5. Discussion

To determine $T_{g}$, both granulate and filament underwent DSC analyses. The PC filament displayed a $T_{g}$ that was 34.2 °C lower than that of the granulate, attributed to additives that improve printability. Consequently, the build chamber temperature $T_{3}$ was already 74.2 °C above the filament’s $T_{g}$, leading to poor dimensional accuracy of PC samples.

The surface roughness analysis showed that sandblasting created a rougher surface on PC than manual sanding, likely due to the fine 180-grit sandpaper used. The G- and C-PC substrates initially have a higher roughness as a result of their fiber reinforcement. The roughness of glass-fiber-reinforced plates decreased with sanding and sandblasting. This might be because the fibers form a harder surface than the PC matrix material, making them less susceptible to abrasion. Carbon fibers, being harder than glass fibers, showed less reduction in roughness from surface treatments.

The dimensional accuracy analysis revealed that the height of all three materials decreased and the base diameter increased with rising temperatures, regardless of surface treatment. The smaller deviations in fiber-reinforced materials can be attributed to the fiber-reinforcement and the lower $T_{g}$ of the PC filament. For PC, the build chamber temperature $T_{1}$ was already 35 °C above the filament’s $T_{g}$, whereas the differences were significantly smaller for G-PC and C-PC.

Torsion tests on PC samples indicated that the interfacial strength increased until a chamber temperature of $T_{2}$. Beyond this critical temperature, both the torsional stress and fracture angle decreased. This suggests that both the thermal behavior of the injection-molded granulate and the filament are crucial for interfacial strength. The high build chamber temperature for the filament caused significant geometric deviations and reduced mechanical properties, likely due to filament degradation. For PC, the sanded samples in a heated chamber consistently performed better than sandblasted ones. Untreated and plasma-treated samples generally showed the highest mechanical properties. Sanding and sandblasting introduced microstructures and irregularities, leading to local stress concentrations and lower mechanical properties. The higher roughness of sandblasted samples compared to sanded ones resulted in lower fracture moments. Plasma treatment tended to increase strength, aligning with findings from Penter et al. [12], by activating the surface and improving wettability, leading to better adhesion.

G-PC samples, similar to PC, showed higher strength in untreated and plasma-treated specimens, with the highest strength achieved at $T_{3}$. However, there were minimal differences between the fracture moments, likely due to similar $R_{a}$ values.

Comparing the results across the three materials, PC samples achieved the highest strength of 39.05 N/mm^2^ with untreated samples printed with chamber temperature $T_{2}$, followed by G-PC samples with a maximum of 38.89 N/mm^2^ for plasma-treated samples printed with $T_{3}$. Neat materials achieved lower maximal torsional stress: 32.11 N/mm^2^ for (plasma-treated and printed with chamber temperature $T_{3}$) and 9.0 Nm for C-PC (plasma-treated and printed with chamber temperature $T_{3}$). The carbon fibers appeared to negatively impact torsional strength. The previous studies by Tekinalp et al. [28] and Liao et al. [29] showed that fibers increase strength in tensile and bending tests when aligned with the printing and loading directions. In this study, the printing direction and, thus, the fiber orientation differed. The filament paths were laid in concentric circles around the hybrid specimen’s longitudinal axis, aligning fibers parallel to the interface and along concentric circles around the longitudinal and torsional axes. This likely resulted in lower fracture moments due to asymmetric stress distributions, as described by Du et al. [30]. Multiple studies have shown that crack propagation in fiber-reinforced plastics under shear and torsional loads mainly occurs through delamination at the fiber–matrix interfaces, explaining the lower torsional strength of carbon-fiber-reinforced composites compared to PC parts.

Furthermore, glass and carbon fibers have higher stiffness and strength than the PC matrix, leading to a more brittle failure behavior of the samples. This behavior was evident in the optical analysis of fracture surfaces. Ductile fractures in PC samples showed significant deformation and crack propagation. In contrast, brittle fractures in fiber-reinforced samples were flatter and lacked visible deformation features. Images of PC samples indicated that the fracture surfaces became more uniform with increasing temperature. However, the highest temperature sample showed irregularities, likely due to filament embrittlement from degradation. The maximal roughness value of the fracture surface increased with temperature across all materials, likely due to more intense diffusion at higher temperatures.

## 6. Conclusions

This study investigated the effects of different surface treatments and build chamber temperatures on the torsional strength of hybrid samples produced through a combination of injection molding and additive manufacturing. The following are the conclusions:

- Among the surface treatments, untreated and plasma-treated samples exhibited the best torsional strength, while abrasive methods like sanding and sandblasting reduced strength. This reduction in strength may be attributed to the formation of stress concentrations from these abrasive treatments, which hinder the diffusion process and consequently lower the torsional strength.
- PC’s ideal build chamber temperature was identified at 166 °C, yielding a maximum torsional strength of 12.3 Nm. The fiber-reinforced samples achieved lower maximum torques of 11.7 Nm for G-PC and 9.0 Nm for C-PC at $T_{3}$. While PC samples displayed ductile behavior with a smooth, spiral fracture pattern at the optimal temperature, fiber-reinforced samples failed abruptly and brittlely without noticeable deformation features.
- Increasing build chamber temperatures led to a decrease in sample height and an increase in base diameter across all materials, affecting the overall dimensional accuracy.
- The fracture surfaces became rougher with increasing temperature for all materials, with PC samples showing a distinctive spiral pattern.

## Figures and Tables

**Table 1:** Printing parameters.

Parameter	Value
Nozzle diameter	0.4 mm
Layer width	0.4 mm
Layer height	0.2 mm
Heatbed temperature	-
Nozzle temperature PC	260 °C
Nozzle temperature G-PC	300 °C
Nozzle temperature C-PC	270 °C

**Table 2:** Glass transition temperature $T_{g}$ of PC, G-PC, and C-PC substrate and filament, respectively, as measured with DSC.

	Substrate	Filament
	$T_{g}$ [°C]	$T_{g}$ [°C]
PC	146	111
G-PC	149	142
C-PC	149	146

**Table 3:** Average ($\bar{x}$) and standard deviation (σ) of torsional stress (τ) and failure angle (α) of PC samples printed at chamber temperatures $T_{0}$–$T_{3}$ and surface treatments no treatment (NT), plasma (PL), sandpaper (SP), and sandblasting (SB).

		$T_{0}$		$T_{1}$		$T_{2}$		$T_{3}$	
		τ	α	τ	α	τ	α	τ	α
		[N/mm^2^]	[°]	[N/mm^2^]	[°]	[N/mm^2^]	[°]	[N/mm^2^]	[°]
NT	$\bar{x}$	4.95	7.61	36.70	32.83	39.05	40.37	25.30	25.37
	σ	1.68	2.73	4.05	4.30	1.73	4.39	5.23	13.52
PL	$\bar{x}$	12.84	16.90	37.16	26.87	37.26	45.69	33.99	39.61
	σ	2.91	3.52	5.12	6.34	3.53	4.50	2.28	7.44
SP	$\bar{x}$	1.96	2.92	37.41	31.83	34.56	37.60	28.01	25.14
	σ	0.78	1.49	1.47	4.00	1.32	5.13	5.28	4.89
SB	$\bar{x}$	3.08	5.26	32.47	28.09	29.13	39.90	24.38	25.14
	σ	2.57	2.02	4.01	6.09	4.57	4.59	6.86	8.63

**Table 4:** Average ($\bar{x}$) and standard deviation (σ) of torsional stress (τ) and failure angle (α) of G-PC samples printed at chamber temperatures $T_{0}$–$T_{3}$ and surface treatments no treatment (NT), plasma (PL), sandpaper (SP), and sandblasting (SB).

		$T_{0}$		$T_{1}$		$T_{2}$		$T_{3}$	
		τ	α	τ	α	τ	α	τ	α
		[N/mm^2^]	[°]	[N/mm^2^]	[°]	[N/mm^2^]	[°]	[N/mm^2^]	[°]
NT	$\bar{x}$	17.46	6.50	36.53	18.12	35.45	20.72	38.51	29.75
	σ	10.71	4.59	4.80	4.72	2.23	4.49	1.61	5.54
PL	$\bar{x}$	18.64	8.39	36.08	16.00	34.34	13.76	38.89	29.93
	σ	5.59	2.97	1.88	2.59	1.50	2.73	2.45	3.64
SP	$\bar{x}$	8.37	4.30	35.99	14.01	35.63	18.70	37.27	31.35
	σ	2.34	1.72	2.18	1.31	2.49	3.26	1.59	2.78
SB	$\bar{x}$	14.07	5.59	31.90	14.68	36.93	18.32	33.72	24.98
	σ	8.83	4.89	9.90	5.91	2.25	1.85	1.64	3.33

**Table 5:** Average ($\bar{x}$) and standard deviation (σ) of torsional stress (τ) and failure angle (α) of C-PC samples printed at chamber temperatures $T_{0}$–$T_{3}$ and surface treatments no treatment (NT), plasma (PL), sandpaper (SP), and sandblasting (SB).

		$T_{0}$		$T_{1}$		$T_{2}$		$T_{3}$	
		τ	α	τ	α	τ	α	τ	α
		[N/mm^2^]	[°]	[N/mm^2^]	[°]	[N/mm^2^]	[°]	[N/mm^2^]	[°]
NT	$\bar{x}$	10.72	10.33	30.27	15.43	30.83	17.43	31.35	22.53
	σ	3.05	3.52	1.83	2.14	3.51	5.48	3.60	3.03
PL	$\bar{x}$	13,67	6.10	31.51	16.23	30.22	17.18	32.11	22.62
	σ	5.11	6.10	1.69	1.77	0.92	2.40	1.27	2.96
SP	$\bar{x}$	7.42	7.54	23.66	16.90	31.03	15.96	24.93	17.26
	σ	4.52	5.38	0.84	1.09	0.60	2.66	2.23	6.01
SB	$\bar{x}$	9.63	2.64	28.61	14.39	30.82	18.97	29.85	22.78
	σ	3.91	0.89	2.64	2.64	2.60	3.58	2.08	3.11

**Table 6:** Maximal roughness value $R_{z}$ of the representative fracture surface of plasma-treated PC, G-PC, and G-PC for chamber temperatures $T_{0}$–$T_{3}$ in μm.

	PC	G-PC	C-PC
$T_{0}$	611μm	285μm	22μm
$T_{1}$	1952μm	608μm	44μm
$T_{2}$	2096μm	634μm	50μm
$T_{3}$	2108μm	642μm	92μm

## Data Availability

Additional data, such as profilometer scans and raw data, are available from the corresponding author upon reasonable request.
